# Bioaccumulation of Metals in Fish Collected from Macapá Urban Aquatic Environments (Brazilian Amazon) and the Risks to Human Health

**DOI:** 10.3390/toxics13020067

**Published:** 2025-01-21

**Authors:** Debora Cristina Damasceno de Souza, Lucilene Finoto Viana, Fábio Kummrow, Claudia Andrea Lima Cardoso, Nathalya Alice de Lima, Izabelle Alexandra Rodrigues Lacerda, Bruno do Amaral Crispim, Alexeia Barufatti, Lúcio André Viana Dias, Alexandro Cezar Florentino

**Affiliations:** 1 Programa de Pós-Graduação em Biodiversidade Tropical (PPGBIO), Universidade Federal do Amapá (UNIFAP), Rod. Juscelino Kubitschek, km 02-Jardim Marco Zero, Macapá 68903-419, AP, Brazil; debora.damas@unifap.br (D.C.D.d.S.); lucviana74@gmail.com (L.A.V.D.); 2 Faculdade de Ciências Exatas e Tecnologia–FACET, Programa de Pós-Graduação em Ciência e Tecnologia Ambiental (PPGCTA), Universidade Federal da Grande Dourados (UFGD), Rod. Dourados Itahum km 12, Dourados 79804-970, MS, Brazil; lucilenefinoto@hotmail.com (L.F.V.); nathalyalima22@gmail.com (N.A.d.L.); 3 Departamento de Ciências Farmacêuticas, Instituto de Ciências Ambientais, Químicas e Farmacêuticas, Universidade Federal de São Paulo (Unifesp)-campus Diadema, Rua São Nicolau, 210-Centro, Diadema 09913-030, SP, Brazil; fkummrow@unifesp.br; 4 Programa de Pós-Graduação em Recursos Naturais (PGRN), Universidade Estadual de Mato Grosso do Sul (UEMS), Rod. Dourados Itahum km 12, Dourados 79804-970, MS, Brazil; claudia@uems.br; 5 Programa de Pós-Graduação em Ciências Ambientais (PPGCA), Departamento de Meio Ambiente e Desenvolvimento (DMAD), Universidade Federal do Amapá (UNIFAP), Rod. Juscelino Kubitschek, km 02-Jardim Marco Zero, Macapá 68903-419, AP, Brazil; izabellaalaacerdaa@gmail.com; 6 Universidade Estadual do Tocantins (UNITINS), Campus Augustinópolis, Rua Planalto, 601, Centro–CEP, Augustinópolis 77960-000, TO, Brazil; bruno.ac@unitins.br; 7 Programa de Pós-Graduação em Biodiversidade e Meio Ambiente (PPGBMA), Faculdade de Ciências Biológicas e Ambientais (FCBA), Universidade Federal da Grande Dourados (UFGD), Rod. Dourados Itahum km 12, Dourados 79804-970, MS, Brazil; alexeiabarufatti@ufgd.edu.br

**Keywords:** metals, bioaccumulation, human health risk assessment, fish contamination, Brazilian Amazon, urban water pollution

## Abstract

Macapá City, located in the Brazilian Amazon, faces critical aquatic pollution challenges due to inadequate sanitation infrastructure, leading to metal contamination in fish within its urban water bodies. Our study evaluated the concentrations of metals (Cu, Cd, Cr, Fe, Mn, Ni, Pb, Zn, and Hg) in muscle tissues of fish from igarapés, ressaca areas, and canals. Samples were collected from six sampling sites to investigate the bioaccumulation of these metals and their potential human health risks. All metals were quantified by atomic absorption spectrometry, except Hg, which was quantified by inductively coupled plasma optical emission spectrometry. Metal concentrations were determined in three carnivorous and seven omnivorous fish species. Cd concentrations exceeded the Brazilian maximum limit established for human consumption in all fish species evaluated. The estimated daily intake (EDI) of Pb and Hg exceeded their reference doses. Our risk assessment, which combined the risk quotient (RQ) for individual metals and the risk index (RI) for metal mixtures, indicated health risks associated with the consumption of fish collected from the study areas. These results demonstrated a worrying exposure to metals (mainly Cd, Pb, and Hg), highlighting the need for environmental management measures and continuous monitoring to protect public health in vulnerable urban areas.

## 1. Introduction

Macapá is the capital of the Brazilian State of Amapá, has an estimated population of 522,357 inhabitants, a territory of 6,563,849 km^2^ [1], and in the Basic Sanitation Ranking of the 100 largest Brazilian cities occupies last place [2]. The Macapá urban area is located on the left bank of the Amazon River’s mouth, inserted in the estuarine coastal zone, and is subject to constant anthropogenic pressure on the use and conservation of soil, water resources, and the climate [3,4]. Its complex urban design is characterized by dry land and flooded areas that interact and are linked, making it in many cases difficult to distinguish where one type of territory begins and where the other ends [5]. Streams (called igarapés), floodplain lakes (called ressaca areas), and channels constitute the extensive network that makes up the system of Macapá urban water bodies [6]. The ressaca areas are under the strong urbanization processes characterized by the presence of wooden houses supported by stilts (called palafitas in Portuguese) built over the waters [3,5]. Approximately 30% of the urban population of Macapá lives in areas with high levels of ressaca. These areas are highly vulnerable to socio-environmental issues and experience a population growth rate of 20% every four years. In general, solid, and liquid domestic wastes generated in stilt houses are released directly into the water below them without any type of treatment [5]. Igarapés are small branches of rivers that generally have preserved riparian forests. The igarapés are used for fishing, leisure, and the transport of people and various products. Channels are the water bodies that cover most of the Macaca urban area and flow directly into the Amazon River. All these aquatic environments are strongly impacted by human activities, the periodic inflows of the Amazon River, and the effect of the tides of the Atlantic Ocean [3]. When considered together, these urban aquatic environments act as natural filters that receive, retain, and attenuate urban drainage [6].

Urban aquatic environments are among the ecosystems most affected by different human activities since cities generate large amounts of liquid and solid waste that are often released directly into local water resources [7,8,9]. Particularly, urban Amazonian aquatic ecosystems are increasingly vulnerable to stress resulting from human activities [3,10], mainly because the sanitation infrastructure has not kept pace with population growth and the speed of urbanization [11]. Therefore, solid and liquid waste from industrial and domestic origins are the main drivers of pollution in these aquatic environments. Notably, these wastes carry potentially toxic organic and inorganic chemicals, including metals, directly into waters [12,13].

Contamination of aquatic environments by toxic metals is one of the most serious environmental problems worldwide, both for the conservation of fish species and for human health due to their bioaccumulation and biomagnification potentials [3,14,15]. Freshwater fish are the main source of protein for Amazonian communities, and the artisanal fishing industry provides the livelihood for around 40% of the families in fishing communities [16]. Although these populations are still little-studied, Rivero et al. [17] demonstrated that fishing families living in Amazon urban areas are highly dependent on fish consumption and use fishing as a subsistence strategy to deal with food insecurity. Approximately 80% of fishing families living in Amazonian urban centers eat fish almost every day [17]. However, studies carried out in different river basins in the State of Amapá have demonstrated the bioaccumulation of toxic metals in the muscle tissue of several fish species, in addition to the risks to human health resulting from fish consumption [18,19,20,21,22]. Thus, the high consumption of fish contaminated by toxic metals from Amazonian rivers can represent serious risks to human health for local vulnerable populations [17,21,23].

Particularly, the most vulnerable population from the Macapá urban area fishes and consumes fish caught in urban water bodies on a daily basis. Considering that small-scale urban fisheries are largely unstudied, despite their enormous relevance for poverty reduction and the development of policies to combat food insecurity [17], our objective was to quantify the concentrations of metals (Cu, Cd, Cr, Fe, Mn, Ni, Pb, Zn, and Hg) in muscle tissue samples from several fish species collected in three distinct aquatic environments (streams, backwater areas, and canals) located in the Macapá urban area. In addition, we assessed the risks to human health of individuals and mixtures of metals bioaccumulated in the muscle tissue of fish samples resulting from fish consumption.

## 2. Materials and Methods

### 2.1. Sampling Sites

**Figure 1:**
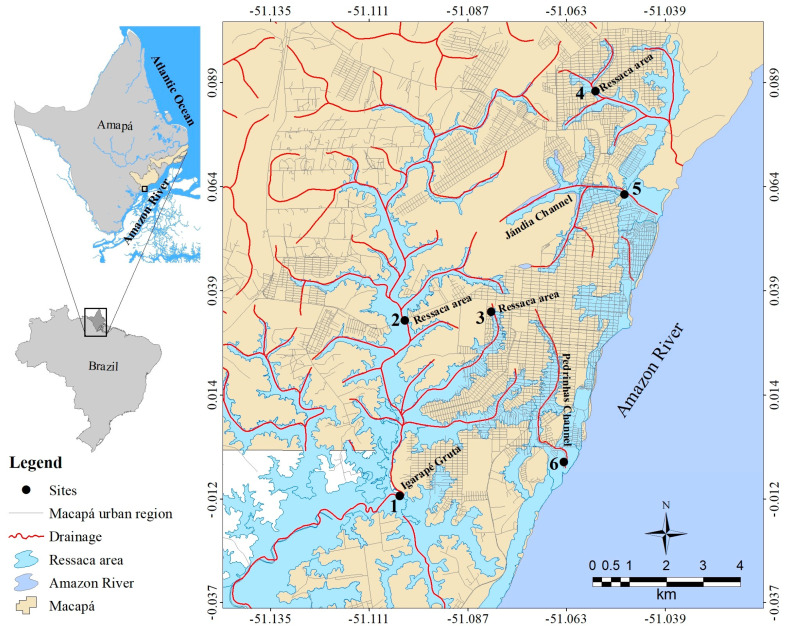
The six sampling sites located in different Macapá urban aquatic environments, Amapá State, Brazil.

For fish sampling, we selected 6 sampling sites located in the Macapá urban area. All sampling sites belong to the Igarapé da Fortaleza sub-basin, which is part of the Amazon River Basin. Three sampling sites are located in channels, two in igarapes, and one in a ressaca area (Figure 1).

Sampling sites were selected based on the presence of strong human influence, particularly the disposal of untreated domestic sewage and solid waste, which negatively impacts both the quality of the waters that receive these wastes and the waters of the Amazon River where urban water bodies flow into. The Amazon River has become the main recipient of industrial effluents, often inadequately treated, from urban regions of the Amapá State [3]. Fish sampling occurred during the day between March and June 2019.

### 2.2. Fish Sampling

All fish samples were collected using gill nets with mesh sizes ranging from 1.5 to 8.0 cm between adjacent nodes and cast nets. After capture, biometric data including total weight (g) and standard length (mm) were obtained using an ichthyometer and a field scale, respectively. In the field, the fish were euthanized by cervical transection, individually labeled, and transported in ice boxes to the laboratory. In the laboratory, fish samples’ taxonomic identification was performed using the specialized literature [24,25]. Specimens were thoroughly rinsed with running water to eliminate surface impurities before tissue dissection. For metal analyses, aliquots of approximately 5 mg of muscle tissue from each sampled fish were collected between the dorsal fin and the end of the caudal peduncle. In the laboratory, samples were kept at −20 °C for a maximum period of 30 days. This project was approved by the Ethics Committee on Animal Use at UNIFAP (016/2019).

### 2.3. Preparation of Fish Muscle Samples and Determination of Metals

The fish muscle tissue samples’ preparation for metal analysis was performed according to the protocol described by Viana et al. [26]. Briefly, muscle samples were dehydrated at 40 °C for 3 h and then macerated and sieved. Aliquots of 0.5 g were transferred to digestion tubes containing 10 mL of a sulfonitric mixture (HNO_3_/H_2_SO_4_; 1:1 *v*/*v*) and V_2_O_5_ 0.1% (*w*/*v*), and then kept at rest for 2 h. Blank samples were prepared according to Olmedo et al. [27]. The samples were analyzed in duplicate using an atomic absorption spectrometer (Shimadzu, model AA7000, Kyoto, Japan), with flame atomization, to quantify Cd, Pb, total Cr, Ni, Fe, Mn, Cu, and Zn [28]. To quantify the Hg present in the samples, we used a hydride generator coupled with inductively coupled plasma optical emission spectrometry. Each metal was measured according to its calibration curves, confirming linearity, a key validation parameter. The operating conditions of the instrument were as follows: power, 1000 W; 15 L min^−1^ of argon gas flow; 1.5 L min^−1^ of auxiliary gas flow; 10 s replication time; 15 s stabilization time; 10 s cleaning time; and a 253 to 652 nm wavelength reading for Hg [26]. Detection limits (µg g^−1^) were as follows: Cu = 0.06; Cd = 0.01; Cr = 0.01; Fe = 0.05; Mn = 0.03; Ni = 0.04; Pb = 0.06; Zn = 0.08; and Hg = 0.10. All analytical standards used were purchased from Merck KGaA, Darmstadt, Germany.

### 2.4. Risk Assessment for Human Health from Fish Consumption and Estimated Daily Intake (EDI)

We performed risk assessments for each metal individually and also for mixtures of metals present in fish muscle tissue samples. For each metal’s risk assessment, we use the risk quotient (RQ) approach. The RQ values were calculated by the ratios between the concentrations of each metal present in the fish muscle tissue samples and the maximum limits (MLs) established by the Brazilian legislation for human consumption [29,30]. RQ values < 1 indicate no risk to human health, while RQ values > 1 indicate risks of adverse effects [31,32]. For the risk assessment of metal mixtures, we use the risk index (RI) approach. The RIs were obtained by summing the RQ values obtained for each metal. The higher the RI value, the greater the risk of damage to human health resulting from fish consumption [21].

To refine risk assessment for human health, we included EDI calculated by the ratio between the concentration of each bioaccumulated metal in the muscle of fish samples and the average daily fish consumption by adult individuals as a function of average body weight. The EDI calculation allows for a more realistic risk estimation since it is based on the concentrations of metals found in food and the best available data on food intake for a specific population [33]. EDI calculation was performed according to the protocol described by Viana et al. [21], as follows:

EDI = BC × Df/BW

where EDI is µg kgbw^−1^ day^−1^; BC is the mean metal concentration bioaccumulated in fish muscle tissue (µg g^−1^); Df is the daily fish consumption rate (416.39 g person^−1^ day^−1^) for the Brazilian Amazon population [34,35]; BW is the average human body weight (60 kg) [34]. The EDI values obtained were compared with reference doses (RfDs) established for each metal. We used the RfDs established by Agência Nacional de Vigilância Sanitália do Brasil, Nota Técnica 8/2019 (ANVISA) [36] for all metals, except for Pb. For Pb, the RfD was proposed by the FAO/WHO [37]. The RfD represents the maximum amount of exposure to each metal that humans can be exposed to without adverse health effects [20,38].

## 3. Results

Nine native fish species were collected from different urban aquatic environments. The non-native species *Oreochromis niloticus* (Linnaeus 1758) was collected from the sampling site located in the ressaca area (sampling site 3). Three species of carnivorous fish (*Acestrorhynchus altus* (Menezes 1969); *Pygocentrus nattereri* (Kner 1858); and *Serrasalmus spilopleura* (Kner 1858)), and seven species of omnivorous fish (*Astyanax lacustris* (Lutken 1875); *Acaronia nassa* (Heckel 1840); *Cichlasoma amazonarum* (Kullander 1983); *Krobia guianensis* (Regan 1905); *Leporinus friderici* (Bloch 1794); *Myloplus rubripinnis* (Müller and Troschel 1844); and *O. niloticus*) were collected. Most fish samples were collected at the sampling site located in the ressaca area (57 specimens), followed by sampling sites located in canals (19 specimens), and in igarapés (17 specimens, all the same species). The weight of the fish samples ranged from 6.00 to 512.57 g and the size from 4.5 to 23.28 cm (Table 1).

### 3.1. Metal Concentrations in Fish Species and Their Compliance with Legal Limits

**Figure 2:**
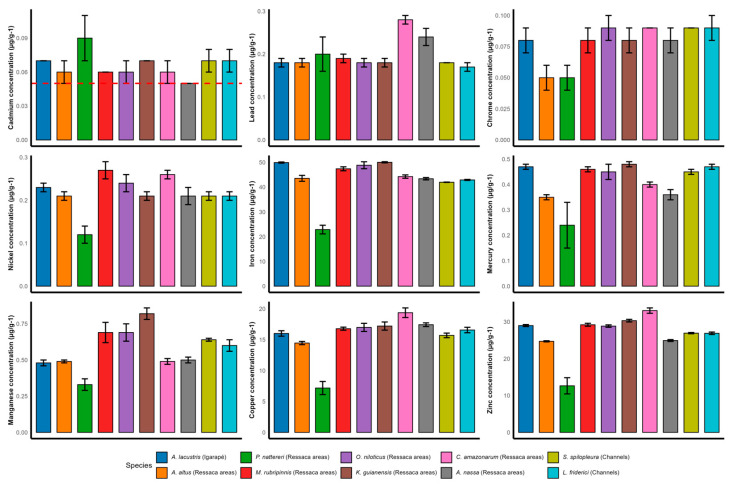
Metal concentrations (µg g^−1^) present in fish muscle samples collected from different Macapá urban aquatic environments. The red dotted line represents the Brazilian maximum limit for Cd [29].

Relatively similar concentrations of the analyzed metals were found in carnivorous and omnivorous fish species collected in different types from the Macapá urban aquatic environments. The concentrations of Pb, Cr, Ni, Hg, Cu, and Zn found in the muscle tissue of fish samples were lower than the respective Brazilian MLs [29,30,36] (Figure 2). Brazilian legislation does not establish an ML for Fe. Cd showed concentrations above the Brazilian ML [29], making all fish species sampled unfit for human consumption, regardless of eating habits, size, weight, foraging behavior, or type of urban aquatic environment from which they were collected (Figure 2). Cd presented an average concentration of ~ 0.06 (µg g^−1^). The fish species that presented higher Cd concentrations in their muscle tissue were *A. nassa* (0.09 µg g^−1^) followed by *A. altus* (0.07 µg g^−1^), *L. friderici* (0.07 µg g^−1^), *S. spilopleura* (0.07 µg g^−1^), *P. nattereri* (0.07 µg g^−1^), *C. amazonarum* (0.06 µg g^−1^), *K. guianensis* (0.06 µg g^−1^), *A. lacustris* (0.06 µg g^−1^), *M. rubripinnis* (0.06 µg g^−1^), and *O. niloticus* (0.05 µg g^−1^). The species with higher Pb concentrations in their muscle tissue were *M. rubripinnis* with ~0.28 µg g^−1^, followed by *O. niloticus* with ~ 0.24 µg g^−1^. *A. nassa* presented the smallest concentrations of Cr, Ni, Fe, Hg, Mn, Cu, and Zn in muscle tissue samples, while *L. friderici* had higher concentrations of Fe, Hg, and Mn (Figure 2).

### 3.2. Human Health Risk Assessment from Fish Consumption

In all fish species sampled, Cd was the only metal that presented risks to human health (RQs > 1). The highest risk was observed for *A. nassa* samples collected at sampling site 4 (ressaca area). Pb, Cr, Ni, Hg, Cu, and Zn did not individually pose risks to human health associated with fish consumption (RQs < 1) for all species sampled from different aquatic environments (Table 2).

**Figure 3:**
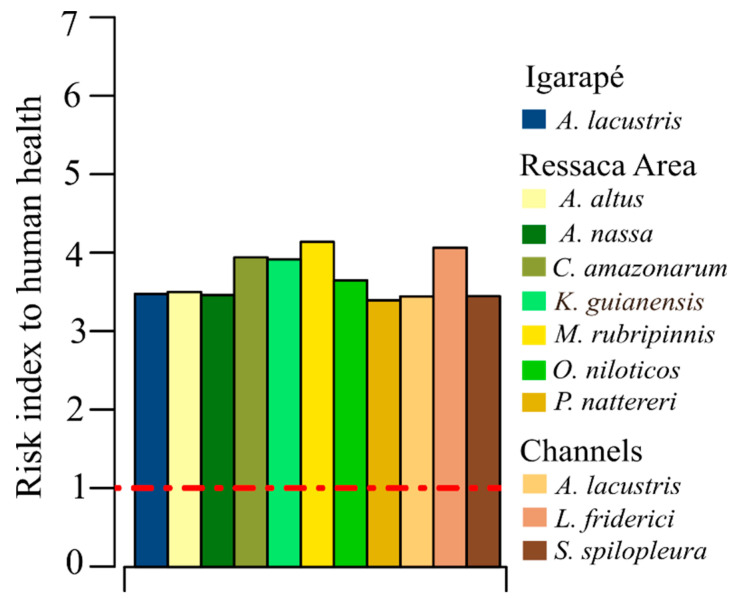
Risk indices (RIs) obtained for the mixtures of metals present in the muscle tissue from fish samples collected in the different Macapá urban aquatic environments. RIs above the red dotted line represent risks to human health (RIs > 1).

All fish species collected presented an RI > 1, indicating a potential risk to human health related to the consumption of these fish (Figure 3).

### 3.3. Estimation of Daily Intake (EDI)

The EDI values obtained for Cd, Cr, Ni, Fe, Mn, and Cu showed lower values than the established RfDs [36]. For all evaluated fish species, except for *S. spilopleura*, the EDI values for Pb exceeded its RfD [37]. Hg presented EDI values higher than its RfD [36] for all fish species evaluated, regardless of the sampling site. This indicates that Pb and Hg pose risks to human health from the daily consumption of these fish (Table 3).

## 4. Discussion

In general, carnivorous fish are considered to have higher concentrations of bioaccumulated metals in their organs and tissues because these species occupy the top of the food chain; however, studies have observed the highest bioaccumulation of metals in omnivorous fish [14]. Viana et al. [21] observed that both the distribution and concentrations of Cr, Ni, Fe, Hg, Mn, Cu, and Zn were similar in all fish species collected in the Araguari River’s lower section (Brazilian State of Amapá), regardless of their feeding habits. Naka et al. [39] pointed out that the increase in Cd in urban Amazonian aquatic environments is mainly related to the irregular disposal of household/urban waste and the burning of fossil fuels used by boats. According to Rico et al. [40], about 90% of wastewater from urban areas located in the Amazon region is discharged without adequate treatment, and it is directly released into the Amazon River or its small tributaries. This wastewater contains several types of toxic chemicals, including different metals [40]. Thus, the bioaccumulation of Cd in muscle tissue from the collected fish samples seems to be related mainly to wastewaters and solid wastes that directly impact urban waterbodies.

Similarly, studies conducted in urban rivers in Bangladesh, such as that by Islam et al. [41], reported metal contamination in both water and fish from the same sites. This contamination was mainly attributed to the discharge of untreated industrial effluents and domestic sewage [41]. In urban rivers in India, elevated concentrations of metals such as Pb and Cd have been associated with the discharge of industrial effluents and agricultural runoff [42,43]. These studies identify Pb and Cd as the main toxic agents in urban water bodies, posing substantial health risks to communities that depend on fish as their main source of protein. This evidence demonstrates that metal contamination is not a problem unique to Macapá but rather a widespread problem affecting urban aquatic ecosystems globally.

In Macapá, fish is commonly consumed as an essential and easily accessible source of protein since urban aquatic environments are located close to human habitations, or even below them, as in the case of the inhabited ressaca areas. Cd levels can vary between species, within species, and by location [44]; however, based on the descriptive analysis of the collected data (Figure 2), no noteworthy variation in Cd concentrations was observed across species, within species, or by location. Diet is the main source of exposure to Cd for the non-smoking population [45], and daily ingestion of fish containing high levels of Cd can cause severe chronic human health problems, including anemia, insomnia, kidney and liver damage, cancers, and osteoporosis, among others [46,47].

However, although the individual concentrations of the analyzed metals do not present risks to human health, with the exception of Cd, it is important to emphasize that chronic exposure to mixtures of different metals can cause oxidative stress, cytotoxicity, immunotoxicity, hepatotoxicity, nephrotoxicity, neurotoxicity, and the development of different types of cancer [12,48]. Therefore, for the risk assessment of mixtures of the different metals present in the muscle tissue samples from each sampled fish species, we calculated IRs. IRs were calculated based on the concentration addition (CA) model [49], commonly used for risk assessment of toxic metal mixtures. The CA model is based on dilution theory and assumes that each constituent of a mixture can be replaced by an effective concentration of another constituent, maintaining the final effect of the mixture [48]. According to Martin et al. [50], the CA model can be safely used as the standard concept for anticipating the combined effects of chemicals. Particularly in the case of mixtures of metal ions, the frequency of synergistic effects is very low and, when they occurred, the concentrations of the metal ions were very high [51].

Throughout the Amazon region, fish is the main source of protein and subsistence for riverine and indigenous communities [11,52], and also for vulnerable urban populations [17]. In this region, the average per capita consumption of fish is 135 kg/person/year, which is considered to be higher than the world average [20]. Thus, this risk assessment of metal mixtures in fish muscle tissue is particularly important in the Macapá urban area because many families consume contaminated fish on a daily basis [17], putting them at serious risk. In addition, metals such as Cd, Pb, Hg, and Cr are also harmful to aquatic biota—even when present in aquatic environments in low concentrations—owing to their high toxicity and bioaccumulation potentials [26] that can compromise biodiversity and regional fisheries’ resources.

The RfD value is the maximum amount considered safe for human exposure by all routes and considering all sources. When a single source exceeds the RfD value, a situation of serious risk may occur, as the population may be exposed to Hg and Pb from sources other than fish, including water, air, and other foods that make up their diet. Hg is a very toxic element, even at low concentrations, and it can induce severe damage to human health, including neurological, mutagenic, carcinogenic, and hepatic damage, among others [21]. Pb is another metal considered highly toxic and is associated with several health problems, including mortality, neurodevelopmental effects, impaired renal function, hypertension, impaired fertility, and adverse pregnancy outcomes. Thus, the FAO/WHO have not formally established an RfD for Pb but suggest that the exposure level under 1.2 µg kgbw^−1^ day^−1^ represents a negligible health risk [37]. However, families residing in areas of urban aquatic environments in Macapá consume these contaminated fish due to the exigencies of their socioeconomic situation, which does not allow for greater diversification of protein sources. Therefore, contamination of freshwater fish by Hg and Pb in the Amazon region threatens food safety and has become a serious public health problem [16,53].

Contamination of water and fish by Hg in the Amazon region is associated with small-scale artisanal gold mining, often carried out illegally, as Hg is used to extract gold from rocks through the amalgamation process [23,53]. The different urban aquatic environments in Macapá are contaminated with Hg, probably originating from sources located long distances away and introduced into the urban environment mainly by the effect of the tide, the Amazon River, or by the atmospheric deposition of particles [18,53]. With the effect of the tide, the contaminants are distributed to other bodies of water, as is the case for all urban aquatic environments studied here. Viana et al. [21] found high EDI values for Hg in eleven Amazonian fish species from the Araguari River, ranging from 2.34 to 2.62 μg kgbw^−1^ day^−1^. Costa et al. [20] reported a high risk of Hg present in the muscle tissue of samples of the Amazonian fish *Plagioscion squamosissimus* collected in the middle and lower sections of the Araguari River. Hacon et al. [18] observed high concentrations of Hg in several Amazonian fish species, highlighting the risks of consuming contaminated fish, especially for riverine and indigenous communities. Viana et al. [13] also found Hg contamination in different organs of a fish species endemic to the Amazon Basin, *Colomesus asellus*, sampled in the Canal das Pedrinhas, which is located on the banks of the Amazon River in the urban area of Macapá.

Contamination of Amazonian aquatic environments by Pb is also associated with mining activities [54], mainly cassiterite mining [55]. Thus, Pb present in water and sediments has been bioaccumulating in several fish species, which poses risks to human health. Other studies have also demonstrated Pb intake via fish consumption above its RfD. EDI values for Pb higher than its RfD were found in muscle tissue samples of the fish species *Triportheus auritus* and *Curimata incompta* collected in the Araguari River lower section, Amapá State [21]. For the species *Ageneiosus inermis* and *Hoplias Aimara*, collected in the upper and middle sections of the Araguari River, Viana et al. [22] obtained Pb EDI values above its RfD; both fish species are among the most captured, traded, and consumed in this region [22]. Taken together, these results are a cause for great concern since fish consumption throughout the Amazon region, including Amapá State, is very high.

### Limitations and Future Research

This study offers critical insights into the bioaccumulation of metals in fish species from urban aquatic environments in Macapá, Brazil, and highlights potential risks to human health. However, certain limitations must be considered to contextualize the findings and guide future research. The sampling sites were confined to urban areas, potentially limiting the representation of metal contamination across the broader Amazon Basin. Expanding the geographic coverage could uncover regional variations and enhance generalizability. Additionally, the temporal dynamics of metal bioaccumulation were not explored; incorporating seasonal sampling would clarify the influence of the Amazonian hydrological cycle on contamination levels. The risk assessment relied on reference doses that may not fully account for chronic, cumulative, or interactive effects of metal mixtures, underscoring the need for advanced toxicological modeling. Furthermore, the absence of direct biomonitoring data from the local population limits the understanding of actual exposure levels. Future research should broaden spatial and temporal sampling, assess toxicological interactions of metals, integrate biomonitoring, and develop strategies to mitigate contamination. Addressing these gaps will refine risk assessments, inform policies, and safeguard public health and biodiversity in the Amazon.

## 5. Conclusions

Metal concentrations and their distribution profiles were similar among different fish species, regardless of their feeding behavior or the type of aquatic environment where they were collected. The IRs obtained for all sampled fish species indicate that the mixture of metals Cd, Hg, Pb, Cr, Ni, Fe, Mn, and Cu found in muscle tissues presented risks related to their consumption. Furthermore, for Pb and Hg, EDI values equal to or greater than their RfDs were found for all fish species. Together, our results indicate that all populations consuming fish caught in aquatic environments from the Macapá urban area are at risk and may develop serious chronic health problems resulting from long-term exposure to metals. Therefore, the urban aquatic environments of Macapá require pollution recovery projects to guarantee the maintenance and conservation of native Amazonian fish species as well as food security.

## Figures and Tables

**Table 1:** Sampled fish species’ length (cm) and weight (g) (Mean ± SD), feeding habits, and habitats in different Macapá urban aquatic environments, Amapá State, Brazil.

Fish Species	Igarapé	Ressaca Areas			Channels		Standard Length (cm)	Total Weight (g)	Feeding Habits	Habitats
	Site 1	Site 2	Site 3	Site 4	Site 5	Site 6				
*A. altus*	0	5	0	0	0	0	11.02 ± 0.72	18.18 ± 3.41	Carnivore	Benthopelagic
*P. nattereri*	0	5	0	0	0	0	8.84 ± 1.66	37.56 ± 20.76	Carnivore	Pelagic
*S. spilopleura*	0	0	0	0	0	4	7.25 ± 0.31	12.80 ± 2.20	Carnivore	Benthopelagic
*A. lacustris*	17	0	0	0	5	0	8.50 ± 0.85	20.01 ± 6.75	Omnivore	Benthopelagic
*A. nassa*	0	0	0	3	0	0	4.50 ± 1.13	6.00 ± 4.24	Omnivore	Benthopelagic
*C. amazonarum*	0	0	7	0	0	0	10.10 ± 0.88	61.62 ± 27.58	Omnivore	Benthopelagic
*K. guianensis*	0	0	20	0	0	0	11.16 ± 0.99	63.80 ± 40.54	Omnivore	Benthopelagic
*L. friderici*	0	0	0	0	0	9	9.02 ± 2.67	23.87 ± 14.82	Omnivore	Benthopelagic
*M. rubripinnis*	0	10	0	0	0	0	9.55 ± 1.66	42.21 ± 15.40	Omnivore	Benthopelagic
*O. niloticus*	0	0	7	0	0	0	23.28 ± 2.46	512.57 ± 166.44	Omnivore	Benthopelagic
Total	17	20	34	3	5	14				

**Table 2:** Risk assessment for human health related to fish consumption sampled in different aquatic environments located within the Macapá urban area. Risk to human health (RQs > 1) is highlighted, representing potential risks as indicated by values above the red dotted line.

		Risk Quotient (RQ)						
Fish Species	Sites	Cd	Pb	Cr	Ni	Hg	Cu	Zn
*A. lacustris*	Igarapé	**1.22**	0.61	0.54	0.04	0.69	0.48	0.49
*A. altus*	Ressaca areas	**1.37**	0.60	0.84	0.04	0.47	0.53	0.58
*A. nassa*	Ressaca areas	**1.88**	0.68	0.48	0.02	0.47	0.24	0.25
*C. amazonarum*	Ressaca areas	**1.26**	0.62	0.84	0.05	0.91	0.56	0.58
*K. guianensis*	Ressaca areas	**1.25**	0.60	0.88	0.05	0.89	0.56	0.57
*M. rubripinnis*	Ressaca areas	**1.12**	0.92	0.90	0.05	0.79	0.64	0.66
*O. niloticus*	Ressaca areas	**1.05**	0.79	0.81	0.04	0.71	0.58	0.50
*P. nattereri*	Ressaca areas	**1.33**	0.60	0.90	0.04	0.45	0.52	0.54
*A. lacustris*	Channels	**1.20**	0.61	0.54	0.04	0.69	0.49	0.49
*L. friderici*	Channels	**1.35**	0.60	0.82	0.04	0.96	0.57	0.60
*S. spilopleura*	Channels	**1.35**	0.57	0.90	0.04	0.47	0.55	0.54

Bold: Cd RQs (>1) represent risks to human health.

**Table 3:** Average daily intake (EDI) (μg kg^−1^ bw day^−1^) of metals through consumption of fish muscle tissue from different species and feeding behavior collected in different urban aquatic environments in Macapá, Amapá, Brazil, and the oral reference dose (RfD) for each metal.

Fish Species	Sites	Estimated Daily Intake (EDI)								
		Cd	Pb	Cr	Ni	Fe	Hg	Mn	Cu	Zn
*A. lacustris*	Igarapé	0.42	**1.28**	0.37	1.43	304.34	**2.41**	3.41	99.75	171.59
*A. altus*	Ressaca areas	0.47	**1.26**	0.58	1.58	346.72	**3.25**	3.36	111.07	201.17
*A. nassa*	Ressaca areas	0.65	**1.41**	0.33	0.80	158.80	**1.64**	2.28	49.86	87.75
*C. amazonarum*	Ressaca areas	0.44	**1.29**	0.58	1.87	329.23	**3.17**	4.78	116.48	202.67
*K. guianensis*	Ressaca areas	0.43	**1.25**	0.61	1.70	339.20	**3.09**	4.80	117.90	200.12
*M. rubripinnis*	Ressaca areas	0.39	**1.92**	9.22	1.84	307.36	**2.74**	3.37	134.44	229.37
*O. niloticus*	Ressaca areas	0.36	**1.65**	0.56	1.48	301.30	**2.48**	3.48	120.93	172.91
*P. nattereri*	Ressaca areas	0.46	**1.25**	0.62	1.48	291.54	**3.15**	4.44	108.93	186.86
*A. lacustris*	Channels	0.41	**1.27**	0.37	1.41	298.72	**2.39**	3.36	101.47	171.37
*L. friderici*	Channels	0.47	**1.25**	0.57	1.45	347.55	**3.32**	5.70	119.45	210.41
*S. spilopleura*	Channels	0.47	**1.20**	0.62	1.44	297.96	**3.24**	4.20	114.98	186.63
RfD		0.83 ^a^	1.2 ^b^	45.00 ^a^	1000.00 ^a^	3470.00 ^a^	0.57 ^a^	2300.00 ^a^	6935.00 ^a^	23,500.00 ^a^

Reference dose (RfD): ANVISA [36] ^a^ and the FAO/WHO [37] ^b^; bold: Hg and Pb EDI values above their RfDs [36,37].

## Data Availability

The data supporting the reported results are available in the manuscript, specifically in Table 1, which provides detailed information on the sampled fish species. This includes their length (cm) and weight (g) (Mean ± SD), feeding habits, and habitats in different urban aquatic environments of Macapá, Amapá State, Brazil.

## References

[1] Instituto Brasileiro de Geografia e Estatística (IBGE) Population Estimates, Macapá 2021. https://www.ibge.gov.br/cidades-e-estados/ap/macapa.html.

[2] Santos C.M.B., Nery C.H.S. (2022). Análise do atual sistema de esgotamento sanitário da cidade de Macapá em conjuntura com realização de estudo de caso do sistema de esgoto encontrado no bairro central. Rev. Mult. CEAP.

[3] Rodrigues C.C.S., Santos L.G.G.V., Santos E., Damasceno F.C., Corrêa J.A.M. (2018). Polycyclic aromatic hydrocarbons in sediments of the Amazon River Estuary (Amapá, Northern Brazil): Distribution, sources and potential ecological risk. Mar. Pollut. Bull..

[4] Sousa T.S., Viegas C.J.T., Cunha H.F.A., Cunha A.C.D. (2023). Drainage and preliminary risk of flooding in an urban zone of Eastern Amazon. GEP.

[5] Costa P.C., Samora P. (2023). Formas urbanas para áreas de conflito socioambiental em APP’s: Modelos para os desafios das áreas de ressaca de Macapá-AP. Rev. Morfol. Urbana.

[6] Takiyama L.R., Silva U.R.L., Jimenez E.A., Pereira R.A. (2013). Zoneamento ecológico-econômico urbano das áreas úmidas de Macapá e Santana, Estado do Amapá. OLAM—CiÊNcia Tecnol..

[7] Flores C.A.R., Cunha A.C., Cunha H.F.A. (2022). Modelagem de lixiviados e compostos gerados em sistema de drenagem de aterro controlado de Macapá/Brasil, Rev. Ibero-Am. CiÊNc. Ambient..

[8] Bega J.M.M., Zanetoni Filho J.A., Albertin L.L., Oliveira J.N.D. (2022). Temporal changes in the water quality of urban tropical streams: An approach to daily variation in seasonality. Integr. Environ. Assess. Manag..

[9] Carim M.D.J.V., Torres A.M., Takyiama L.R., Silva Junior O.M.D., Souza M.O.D., Souto F.A.F., Baia M., Barata J.B., Souza A.J.B.D., Correa P.R.D.S. (2022). Impactos da disposição de resíduos sólidos urbanos no solo e água nos municípios de Macapá e Santana—Amapá. RSD.

[10] Albuquerque F.E.A., Herrero-Latorre C., Miranda M., Barrêto Júnior R.A., Oliveira F.L.C., Sucupira M.C.A., Ortolani E.L., Minervino A.H.H., López-Alonso M. (2021). Fish tissues for biomonitoring toxic and essential trace elements in the lower Amazon. Environ. Pollut..

[11] Ferreira M.D.S., Fontes M.P.F., Pacheco A.A., Lima H.N., Santos J.Z.L. (2020). Risk assessment of trace elements pollution of manaus urban rivers. Sci. Total Environ..

[12] Yuan G., Dai S., Yin Z., Lu H., Jia R., Xu J., Song X., Li L., Shu Y., Zhao X. (2014). Toxicological assessment of combined lead and cadmium: Acute and sub-chronic toxicity study in rats. Food Chem. Toxicol..

[13] Viana L.F., Súarez Y.R., Cardoso C.A.L., Crispim B.D.A., Grisolia A.B., Lima-Junior S.E. (2017). Mutagenic and genotoxic effects and metal contaminations in fish of the Amambai River, upper Paraná River, Brazil. Environ. Sci. Pollut. Res..

[14] Ali H., Khan E. (2018). Bioaccumulation of non-essential hazardous heavy metals and metalloids in freshwater fish. risk to human health. Environ. Chem. Lett..

[15] Dagosta F.C.P., Pinna M.D. (2019). The fishes of the Amazon: Distribution and biogeographical patterns, with a comprehensive list of species. Bull. Am. Mus. Nat. Hist..

[16] Albuquerque F.E.A., Minervino A.H.H., Miranda M., Herrero-Latorre C., Barrêto Júnior R.A., Oliveira F.L.C., Sucupira M.C.A., Ortolani E.L., López-Alonso M. (2020). Toxic and essential trace element concentrations in fish species in the lower Amazon, Brazil. Sci. Total Environ..

[17] Rivero S.L.M., Almeida O.T.D., Torres P.C., De Moraes A., Chacón-Montalván E., Parry L. (2022). Urban Amazonians use fishing as a strategy for coping with food insecurity. JDS.

[18] Hacon S.D.S., Oliveira-da-Costa M., Gama C.D.S., Ferreira R., Basta P.C., Schramm A., Yokota D. (2020). Mercury exposure through fish consumption in traditional communities in the Brazilian northern amazon. Int. J. Environ. Res. Public Health.

[19] Viana L.F., Kummrow F., Cardoso C.A.L., Lima N.A., Solórzano J.C.J., Crispim B.A., Barufatti A., Florentino A.C. (2021). High concentrations of metals in the waters from Araguari River lower section (Amazon Biome): Relationship with land use and cover, ecotoxicological effects and risks to aquatic biota. Chemosphere.

[20] Costa M.S., Viana L.F., Cardoso C.A.L., Isacksson E.D.G.S., Silva J.C., Florentino A.C. (2022). Landscape composition and inorganic contaminants in water and muscle tissue of *Plagioscion squamosissimus* in the Araguari River (Amazon, Brazil). Environ. Res..

[21] Viana L.F., Kummrow F., Cardoso C.A.L., Lima N.A., Crispim B.A., Barufatti A., Florentino A.C. (2023). Metal bioaccumulation in fish from the Araguari River (Amazon Biome) and human health risks from fish consumption. Environ. Sci. Pollut. Res..

[22] Viana L.F., Cardoso C.A.L., Oliveira M.S.B., Lima-Junior S.E., Kummrow F., Florentino A.C. (2024). Metals bioaccumulation in fish captured from Araguari River upper section (Amazon Biome), and risk assessment to human health resulting from their consumption. J. Trace Elem. Min..

[23] Martoredjo I., Calvão Santos L.B., Vilhena J.C.E., Rodrigues A.B.L., De Almeida A., Sousa Passos C.J., Florentino A.C. (2024). Trends in mercury contamination distribution among human and animal populations in the amazon region. Toxics.

[24] Santos G.M., Juras A.A., Mérona B., Jégue M. (2004). Peixes do Baixo rio Tocantins. 20 anos Depois da Usina Hidrelétrica Tucuruí.

[25] Sleen P.V., Albert J.S. (2018). Field Guide to the Fishes of the Amazon, Orinoco, and Guianas.

[26] Viana L.F., Cardoso C.A.L., Lima-Junior S.E., Súarez Y.R., Florentino A.C. (2020). Bioaccumulation of metal in liver tissue of fish in response to water toxicity of the Araguari-Amazon River, Brazil. Environ. Monit. Assess..

[27] Olmedo P., Pla A., Hernández A.F., Barbier F., Ayouni L., Gil F. (2013). Determination of toxic elements (mercury, cadmium, lead, tin and arsenic) in fish and shellfish samples: Risk assessment for the consumers. Environ. Int..

[28] Morgano M.A., Gomes P.C., Mantovani D.M.B., Perrone A.A.M., Santos T.F. (2005). Níveis de mercúrio total em peixes de água doce de pisciculturas paulistas. CiÊNc. Tecnol. Aliment..

[29] Agência Nacional de Vigilância Sanitária do Brasil (ANVISA) (1998). Portaria nº 685 de 27 de Agosto de 1998, Brasília. https://www.univates.br/unianalises/media/imagens/Anexo_XI_61948_11.pdf.

[30] Agência Nacional de Vigilância Sanitária do Brasil (ANVISA) (2013). Legislação Brasileira, Resolução nº 42 de 29 de Agosto de 2013, Brasília. https://bvsms.saude.gov.br/bvs/saudelegis/anvisa/2013/rdc0042_29_08_2013.html.

[31] Ullah A.K.M.A., Maksud M.A., Khan S.R., Lutfa L.N., Quraishi S.B. (2017). Development and validation of a gf-aas method and its application for the trace level determination of Pb, Cd, and Cr in fish feed samples commonly used in the hatcheries of Bangladesh. J. Anal. Sci. Technol..

[32] USEPA (2000). Risk Based Concentration Table.

[33] Duffus J.H., Duffus J.H., Nordberg M., Templeton D.M. (2007). Glossary of terms used in toxicology, 2nd edition (IUPAC Recommendations 2007). Pure Appl. Chem..

[34] Isaac V.J., Almeida M.C., Giarrizzo T., Deus C.P., Vale R., Klein G., Begossi A. (2015). Food Consumption as an Indicator of the Conservation of Natural Resources in Riverine Communities of the Brazilian Amazon. An. Acad. Bras. CiÊNc..

[35] Souza-Araujo J.D., Hussey N.E., Hauser-Davis R.A., Rosa A.H., Lima M.D.O., Giarrizzo T. (2022). Human risk assessment of toxic elements (As, Cd, Hg, Pb) in marine fish from the Amazon. Chemosphere.

[36] Agência Nacional de Vigilância Sanitária do Brasil (ANVISA) (2019). Nota Técnica nº 8/2019/SEI/GEARE/GGALI/DIRE2/ ANVISA. Processo nº 25351.918291/2019–53, Avaliação de Risco: Consumo de Pescado Proveniente de Regiões Afetadas Pelo Rompimento da Barragem do Fundão/MG. https://sanityconsultoria.com/wp-content/uploads/2019/06/nota-tecnica-anvisa-pescado-rio-doce-junho-2019.pdf.

[37] FAO/WHO (2011). Evaluation of Certain Food Additives and Contaminants. Seventy-Third Report of the Joint FAO/WHO Expert Committee on Food Additives.

[38] Musarrat M., Ullah A.K.M.A., Moushumi N.S., Akon S., Nahar Q., Saliheen Sultana S.S., Quraishi S.B. (2021). Assessment of heavy metal(loid)s in selected small indigenous species of industrial area origin freshwater fish and potential human health risk implications in Bangladesh. LWT.

[39] Naka K.S., Mendes L.C.S., Queiroz T.K.L., Costa B.N.S., Jesus I.M., Câmara V.M., Lima M.O. (2020). A comparative study of cadmium levels in blood from exposed populations in an industrial area of the Amazon, Brazil. Sci. Total Environ..

[40] Rico A., Oliveira R., Nunes G.S.S., Rizzi C., Villa S., López-Heras I., Vighi M., Waichman A.V. (2021). Pharmaceuticals and other urban contaminants threaten Amazonian freshwater ecosystems. Environ. Int..

[41] Islam M.S., Ahmed M.K., Habibullah-Al-Mamun M., Masunaga S. (2015). Assessment of trace metals in fish species of urban rivers in Bangladesh and health implications. Environ. Toxicol. Pharmacol..

[42] Rizwan K.M., Thirukumaran V., Suresh M. (2021). Assessment and source identification of heavy metal contamination of groundwater using geospatial technology in Gadilam River Basin, Tamil Nadu, India. Appl. Water Sci..

[43] Dhanakumar S., Solaraj G., Mohanraj R. (2015). Heavy metal partitioning in sediments and bioaccumulation in commercial fish species of three major reservoirs of River Cauvery Delta Region, India. Ecotoxicol. Environ. Saf..

[44] Zhang H., Reynolds M. (2019). Cadmium exposure in living organisms: A short review. Sci. Total Environ..

[45] Zhao D., Wang P., Zhao F.-J. (2023). Dietary cadmium exposure, risks to human health and mitigation strategies. Crit. Rev. Environ. Sci. Technol..

[46] Klaassen C.D., Liu J., Diwan B.A. (2009). Metallothionein protection of cadmium toxicity. Toxicol. Appl. Pharmacol..

[47] Witkowska D., Słowik J., Chilicka K. (2021). Heavy metals and human health: Possible exposure pathways and the competition for protein binding sites. Molecules.

[48] Anyanwu B., Ezejiofor A., Igweze Z., Orisakwe O. (2018). Heavy metal mixture exposure and effects in developing nations: An update. Toxics.

[49] Loewe S., Muischnek H. (1926). Über kombinationswirkungen: Mitteilung: Hilfsmittel der fragestellung. Archiv. F. Exp. Pathol. U. Pharmakol..

[50] Martin O., Scholze M., Ermler S., McPhie J., Bopp S.K., Kienzler A., Parissis N., Kortenkamp A. (2021). Ten years of research on synergisms and antagonisms in chemical mixtures: A systematic review and quantitative reappraisal of mixture studies. Environ. Int..

[51] Bureš M.S., Cvetnić M., Miloloža M., Kučić Grgić D., Markić M., Kušić H., Bolanča T., Rogošić M., Ukić Š. (2021). Modeling the toxicity of pollutants mixtures for risk assessment: A review. Environ. Chem. Lett..

[52] Silva S.F., De Oliveira Lima M.O. (2020). Mercury in fish marketed in the Amazon Triple Frontier and health risk assessment. Chemosphere.

[53] Rodriguez-Levy I.E., Van Damme P.A., Carvajal-Vallejos F.M., Bervoets L. (2022). Trace element accumulation in different edible fish species from the Bolivian Amazon and the risk for human consumption. Heliyon.

[54] Waichman A.V., Nunes G.S.S., Oliveira R., Isabel López-Heras I., Rico A. (2025). Human health risks associated to trace elements and metals in commercial fish from the Brazilian Amazon. J. Environ. Sci..

[55] Azevedo S.M., Nascimento L.S., Silva L.O., Almeida M.G., Azevedo L.S., Constantino W.D., Bastos W.R., Pestana I.A. (2023). Flood pulse as a driving force of Pb variation in four fish guilds from Puruzinho Lake (western Amazon). Environ. Sci. Pollut. Res..

